# Liquefied Petroleum Gas Monitoring System Based on Polystyrene Coated Long Period Grating

**DOI:** 10.3390/s18051435

**Published:** 2018-05-05

**Authors:** Flavio Esposito, Aldobenedetto Zotti, Giovanna Palumbo, Simona Zuppolini, Marco Consales, Antonello Cutolo, Anna Borriello, Stefania Campopiano, Mauro Zarrelli, Agostino Iadicicco

**Affiliations:** 1 Department of Engineering, University of Naples “Parthenope”, 80143 Napoli, Italy; flavio.esposito@uniparthenope.it (F.E.); giovanna.palumbo@uniparthenope.it (G.P.); campopiano@uniparthenope.it (S.C.); 2 Institute for Polymers, Composites and Biomaterials (IPCB)-CNR, 80055 Portici, Italy; aldobenedetto.zotti@unina.it (A.Z.); simona.zuppolini@cnr.it (S.Z.); borriell@unina.it (A.B.); 3 Optoelectronics group, Department of Engineering, University of Sannio, 82100 Benevento, Italy; consales@unisannio.it (M.C.); cutolo@unisannio.it (A.C.); 4 Centro Regionale Information Communication Technology-CeRICT scrl, 82100 Benevento, Italy

**Keywords:** chemical sensors, gas detectors, long period gratings, optical fiber sensors, polymers

## Abstract

In this work, we report the in-field demonstration of a liquefied petroleum gas monitoring system based on optical fiber technology. Long-period grating coated with a thin layer of atactic polystyrene (aPS) was employed as a gas sensor, and an array comprising two different fiber Bragg gratings was set for the monitoring of environmental conditions such as temperature and humidity. A custom package was developed for the sensors, ensuring their suitable installation and operation in harsh conditions. The developed system was installed in a real railway location scenario (i.e., a southern Italian operative railway tunnel), and tests were performed to validate the system performances in operational mode. Daytime normal working operations of the railway line and controlled gas expositions, at very low concentrations, were the searched realistic conditions for an out-of-lab validation of the developed system. Encouraging results were obtained with a precise indication of the gas concentration and external conditioning of the sensor.

## 1. Introduction

Hydrocarbons are a very dangerous species, even when considered at low concentrations, due to high flammable and volatile behavior. As their main application is for fuels, their demand is always increasing, which in turn increases their transport frequency on both roads and rails. For the safekeeping of both the environment and public health, a real-time monitoring of leaks during their transportation would be an appealing solution. Typical gas sensors are built on planar architectures and their working principle is based on the changes in electrical properties of specific materials (the most common are metal oxides), when they are exposed to the analyte gas species. One of the most important parameters is the lower explosive limit (LEL) and, as a consequence, the sensors are designed to detect concentrations below this value, which is different for each target. Unfortunately, commercial sensors generally require high operating temperatures and may not be suitable for use in hostile, harsh and remote environments. In addition, they should be able to operate safely even in an explosive atmosphere [1,2].

Currently, fiber optic technology represents a successful alternative, employed for the detection of very small amounts of chemical and gaseous species. In addition, optical fiber sensors have the advantages of small size, immunity to electromagnetic interferences (EMI), and the possibility of both easy multiplexing and long distance monitoring [3,4]. Nowadays, different configurations and sensitive materials have been proposed for their realization. Among these technologies, we recall refractometric and interferometric configurations [5,6,7], ring resonators [8], etched/tilted fiber Bragg gratings (FBGs) [9,10], long-period gratings (LPGs) [11], photonic crystal fibers [12], surface plasmon resonance (SPR) [13,14], and lossy mode resonance (LMR) sensors [14].

Based on the number of studies carried out over the last decades, we believe that LPG technology is one of the most mature in this field. Special designs such as mode transition (MT) and dispersion turning point (DTP) have been investigated to increase their sensitivity as compared to bare gratings [15,16,17,18,19,20]. As a result, these devices have been used for the detection of several solvents, as well as aromatic and aliphatic hydrocarbons. For example, in [21], a LPG operating at DTP with alternate thin films of PAA/PAH was used to detect ammonia. The syndiotactic polystyrene (sPS) was proposed as a high refractive index layer (HRI) for the LPG in order to detect chloroform in water [22]. Metal organic framework thin films were used in [23] to detect methanol, ethanol, isopropanol, and acetone. Different LPGs were used to develop a system for indoor air quality monitoring in [24]. The selective detection of toluene was demonstrated in [25] by using an LPG coated with a calixarene. Whereas, in [26], an LPG was interrogated by ring-down spectroscopy to detect m-xylene, cyclohexane, trichloroethylene and commercial gasoline. Methane detection was achieved in [27] by using a LPG coated with polycarbonate/cryptophane-A HRI overlay, and in [28] through a coated LPG in photonic crystal fiber. Finally, such devices were also used for hydrocarbon detection in fuel, water and atmospheric environment [29,30].

In our previous work [31], we have presented a butane gas sensor based on a single-ended LPG coated with atactic polystyrene (aPS). The aPS was chosen in light of the affinity between its olefin chains and hydrocarbon molecules. Moreover, its thickness was designed in order to work in mode transition when air is the surrounding medium, in order to attain a significant sensitivity of the final device. Finally, the sensor was tested towards butane in a very low concentrations range, 0.1–1.0 vol %, by means of an in-lab procedure involving a gas test chamber.

In this work, expanding on the results of previous characterizations, we report on the demonstration in a real scenario of a monitoring system for liquefied petroleum gas (LP gas), within the Italian funded project OPTOFER “Innovative optoelectronic technologies for the monitoring and diagnostics of the railway infrastructure”. LP gas is one of the dangerous species being transported with higher frequency (first in Italy) by railways lines. Specifically, the system was installed in a railway tunnel ~850 m long, located between Porta Rufina and Arco Traiano Station in Benevento (Italy), whereas the control and interrogation units were hosted in Porta Rufina Station, 300 m away from the tunnel entrance. The location is part of the railway line Avellino–Benevento run by Italian railways, with passenger trains powered by diesel motors. The monitoring system is based on the aPS coated LPG butane (the main component of LP gas) sensor, which is combined with two fiber Bragg grating sensors for the measurement and compensation of environmental temperature and humidity changes. The sensors are housed in a custom package, which was developed in order to ensure the operation in harsh conditions. Finally, the system was tested in different operating states: (i) with the railway line under normal operation; and (ii) under controlled exposition to the target gas.

In Section 2, we describe the development of the gas monitoring system, focusing on the different components. In Section 3, we report on the system installation and show the experimental results obtained during the in-field tests performed at Porta Rufina Station.

## 2. Development of the Gas Monitoring System

**Figure 1:**
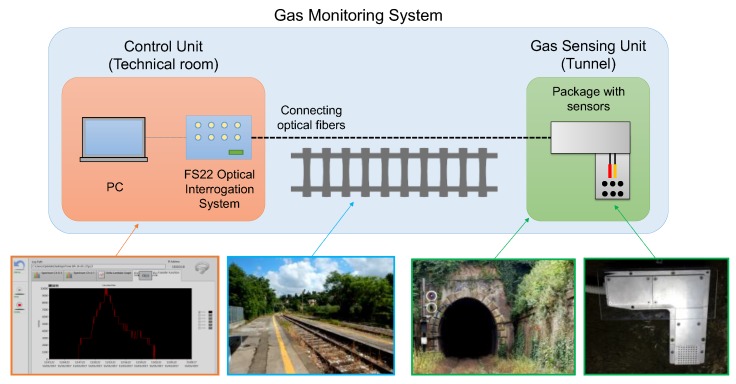
Schematic figure and photos of the gas monitoring system.

The gas monitoring system herein proposed is based on the architecture depicted in Figure 1, where two main components can be distinguished: the sensing unit (SU) and the control unit (CU). The SU is located in the railway tunnel and is composed of an aluminum package, hosting the following fiber sensors: (i) a LPG-based gas sensor and (ii) a FBG-based temperature and humidity sensor. The SU is connected to the CU, located in the technical room of the railway station, by two 500 m long single mode fibers. The CU comprises two main hardware systems, the HBM FS22 optical interrogator and a personal computer, respectively. The interrogator is controlled through a custom LabView software, which manages all the controls and operations, starting from the acquisition of sensors spectra up to the real-time estimation of gas concentration.

In the following, a detailed description concerning the development of each component is reported.

### 2.1. Gas Sensor Based on aPS Coated LPG

**Figure 2:**
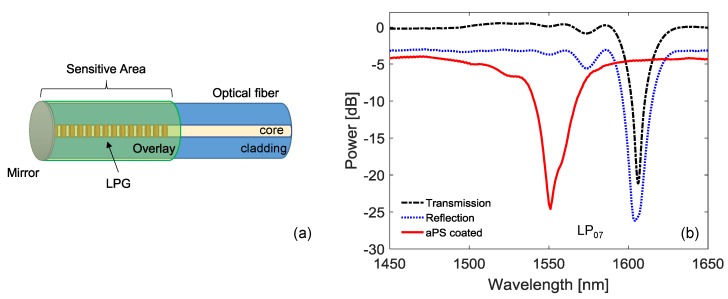
Single ended long-period grating (LPG)-based gas sensor: (**a**) schematic figure; (**b**) power spectrum during the fabrication steps.

The core of the SU consists of the single-ended LPG, coated with a thin layer of aPS as a sensitive overlay, as schematically depicted in Figure 2a. The design and characterization of such a sensor has been presented and discussed in our previous paper [31]. Briefly, we focused the attention on LPG technology, since it has been employed in several physical, chemical, and biological applications [32,33,34,35,36,37,38]. It consists of a periodic perturbation, with period of hundreds of μm, in the refractive index and/or the geometry of the optical fiber [39,40,41].

For the purpose of this work, a commercially available LPG written by UV technique in a standard SMF28 fiber (d_core_ = 8.2 μm, d_clad_ = 125.0 μm, and NA = 0.12) was selected. The LPG was fabricated with a period of Λ = 360 μm which presents attenuation bands related to the coupling with relatively high order cladding modes in the NIR range. In this spectral range, the LP_07_ band was chosen due to the high sensitivity to surrounding refractive index and overlay refractive index. The experimental transmission spectrum of the grating around the band at 1606 nm related to LP_07_ is reported in Figure 2b, black line. We developed a single-ended (or reflection) grating, since this approach allows simpler and strain-free packaging and at same time makes the integration with sensitive layers easier. In particular, for this aim, the fiber was cut along the grating and a silver mirror was deposited on the fiber tip by using the Tollens’ test, according to the procedure reported in [31]. The resulting reflected spectrum of the LPG is reported in Figure 2b with blue line.

For the sensitive material, we used the atactic polystyrene (aPS) due to the chemical affinity between its olefin chains and hydrocarbon molecules [31]. Moreover, the aPS has a refractive index of 1.55, which is higher than silica, and thus its thickness can be designed in order to tune the LPG working point in mode transition region. The design of the aPS overlay optimum thickness was performed by using our numerical tool developed in Matlab environment [42]. The model was particularized by using the parameters reported in [31], finding that aPS thickness has to be selected in the range 320–380 nm, resulting in a wavelength blue shift of the LP_07_ band in range 40–75 nm after the deposition. To this aim, the film was deposited by using the dip-coating technique, starting from a 9.5% *w/w* aPS solution in chloroform at 60 °C (aPS MW = 280.000 g/mol, from Sigma-Aldrich, Milan, Italy). The aPS coated LPG was subjected to a blue shift of 53 nm for LP_07_ band, in good agreement with design criteria, as shown in Figure 2b with the red line. In addition, it should be remarked that our design resulted in the resonance wavelength of the final device being located in the middle of the FS22 system range (1500–1600 nm). Finally, it is worth highlighting that the coated LPG exhibits a very low decrease in the band depth, achieved through the design of the single-ended LPG in over-coupling state.

**Figure 3:**
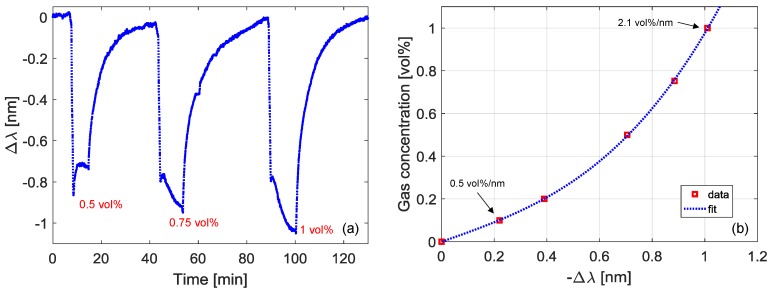
(**a**) Wavelength shift of the LP_07_ band during the exposure to different butane concentrations; (**b**) gas concentration versus (negative) wavelength shift.

The sensor response towards butane was characterized by using a custom-designed test chamber. The set-up is constituted by two lines: the first one for the butane and the second one for an inert gas (nitrogen), and the gas flow is tuned using mass flow controllers. The complete schematic of the experimental setup for gas testing can be found in [31]. The sensor was thus exposed to different concentrations of butane, in the range 0.1–1.0 vol %. As an example, we report in Figure 3a the variation in the spectral position of the LP_07_ band when the LPG was exposed to butane concentrations of 0.5, 0.75, and 1.0 vol %. The sensor practically exhibits an immediate reaction to gas presence, and the following response times were measured: rising T_90_~2–5 min and falling T_10_~15 min. The curve in Figure 3b reports the gas concentration as a function of the negative wavelength shift within the range 0–1.0 vol %. As one can observe, the data fitting can be obtained with a 3rd degree polynomial. Around the concentration of 0.1 vol %, the slope is 0.5 vol %/nm, while it increases to 2.1 vol %/nm around 1.0 vol % of butane. Finally, it should be highlighted that butane LEL is equal to 1.8 vol %, and thus we focused the attention on concentrations lower than about half the LEL.

### 2.2. Gas Sensor Cross Sensitivities to Environmental Conditions

In our previous experimentation [31], tests were performed on an aPS coated LPG sensor under controlled temperature and relative humidity (RH) conditions. In a real scenario, it is necessary to deal with the variations in environmental conditions, especially if operation in harsh environments is envisaged. For this reason, the sensor was characterized towards temperature and humidity changes, considering values being compatible with the selected scenario: 0 °C < T < 15 °C and 75% < RH < 95%.

**Figure 4:**
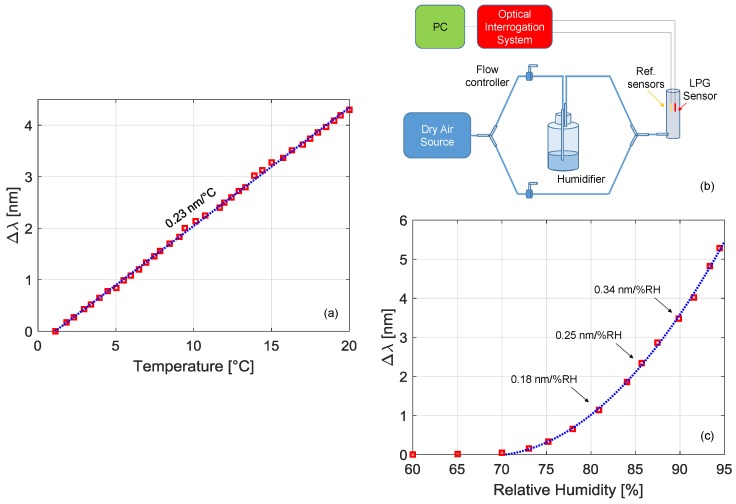
(**a**) Wavelength shift of the LPG sensor versus temperature; (**b**) schematic setup for humidity characterization; (**c**) wavelength shift of the LPG sensor versus humidity.

Concerning the temperature characterization, we used the setup reported in [43,44], which was modified for the housing of single-ended sensors and where a Peltier cell-based system was employed to change the temperature in the range 0–20 °C. The wavelength shifts of the LP_07_ band versus temperature are reported in Figure 4a, where a linear behavior can be appreciated with sensitivity S_T_ = 0.23 nm/°C.

For the humidity characterization, we used the setup depicted in Figure 4b. The setup consists of two lines: the first one is connected to a dry air source (~20% RH), while the second one is connected to a humidifier. Both lines are connected to flow controllers which can be regulated to change humidity from 20% to 95% in the sensor chamber (as measured from reference sensor). The results, in term of the wavelength shift of the LP_07_ band versus humidity level, are reported in Figure 4c. The response is negligible below 70% RH, while it exhibits a quadratic-like behavior for higher humidity conditions, as from the blue fitting curve. For example, within the range 80–90%, the sensitivity S_RH_ increases from 0.18 to 0.34 nm/%RH.

From these results, it is clear that during the in-field testing, a compensation of temperature and humidity changes is necessary. To this end, the well-settled FBG technology was considered in order to monitor real time variations of temperature and humidity level at the installation location. In fact, such devices are normally sensitive to temperature changes, whereas they can be used for humidity sensing when coated with hygroscopic materials by exploiting the strain induced by the latter when subjected to humidity changes [45]. Moreover, their interrogation is also possible by using the same instrumentation used for LPG. 

**Figure 5:**
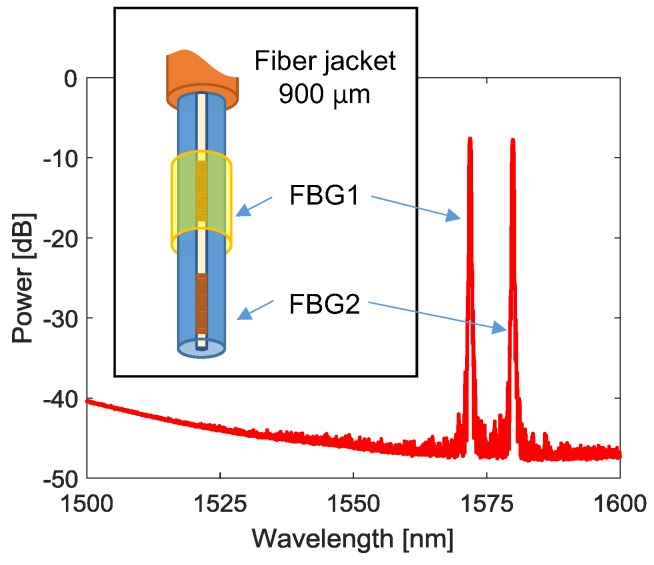
Fiber Bragg gratings (FBG) array for temperature and humidity compensation: schematic and power spectrum.

In particular, we designed a single-ended array composed of two 0.5 cm long FBGs (spaced 1.0 cm), namely FBG1 and FBG2, written in SMF28 fiber, and schematically reported in Figure 5. The first one is coated with a polyimide (PI) layer with thickness around 20 μm and resonance wavelength λ_B1_ = 1572.067 nm. The second one is bare and characterized by λ_B2_ = 1579.95 nm. The resonance wavelengths of the array were designed in order to avoid spectral overlapping even during the sensing phase; in addition, they can be potentially used in multiplexing with the LPG gas sensor. The spectrum of the array is reported in Figure 5 as well. Finally, both FBGs were characterized, resulting in the following sensitivities to temperature—S_T1_ = S_T2_ = 10.1 pm/°C—and humidity—S_RH1_ = 2.8 pm/%RH (FBG2 shows no humidity dependency).

### 2.3. Sensor Packaging

**Figure 6:**
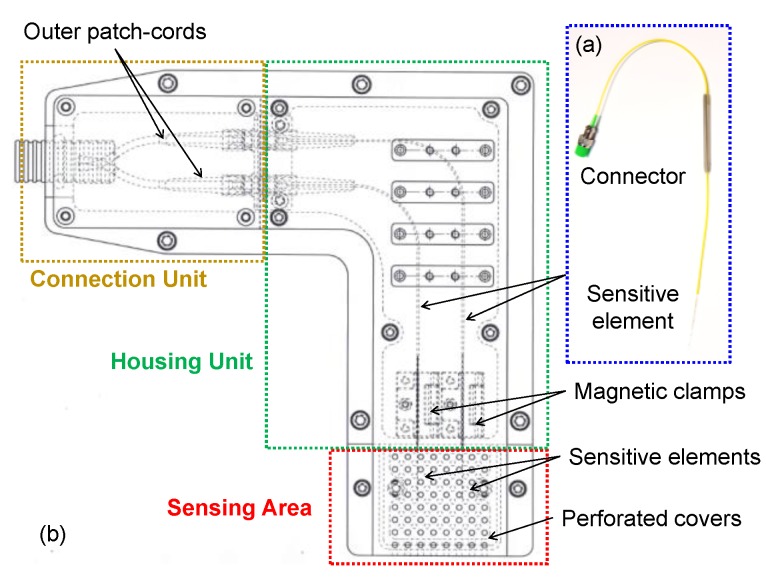
(**a**) Picture of the fiber optic sensor prepared to be installed inside the package; (**b**) CAD image of the designed package.

In order to employ the developed fiber optic sensing system in a real railway location scenario, a custom aluminum package was properly designed and realized to host the two fibers containing both the LPG- and the FBG-based sensors. The package was designed to address the following requirements:

- To enable its fixing on the inner wall of a railway tunnel;
- To protect the fibers (prepared as in Figure 6a) while allowing air exchange with the external environment;
- To keep both fibers in a strain-free state and to avoid their bending, as a consequence of the air motion induced by the train transit;
- To avoid water infiltration inside the package.

Figure 6b reports the CAD image of the designed package. It is mainly composed of three independent regions: (i) the “sensing area”, containing the sensitive area of the fiber devices; (ii) the “housing unit”, where the two optical fibers are positioned and kept fixed by means of properly designed magnetic clamps; and (iii) the “connection unit”, needed to connect the optical fibers containing the sensors with the outer patch-cords travelling throughout the tunnel and reaching the technical room where the interrogation system is located.

To protect the fiber optic sensors as much as possible during train transit inside the tunnel, the “sensing area” was designed so as to be covered with two independent perforated covers, put one on the other, with holes having a diameter of 2.5 mm. The holes of the two covers were designed in such a way to be offset one from the other (i.e., to create a “labyrinth” type path) in order to avoid the air movement during train transit being able to enter directly into contact with the sensors. In this way, indeed, the air is forced to follow the labyrinth path, thus slowing down after the impact with the undrilled part of the cover before coming into contact with the sensitive area of the sensors.

**Figure 7:**
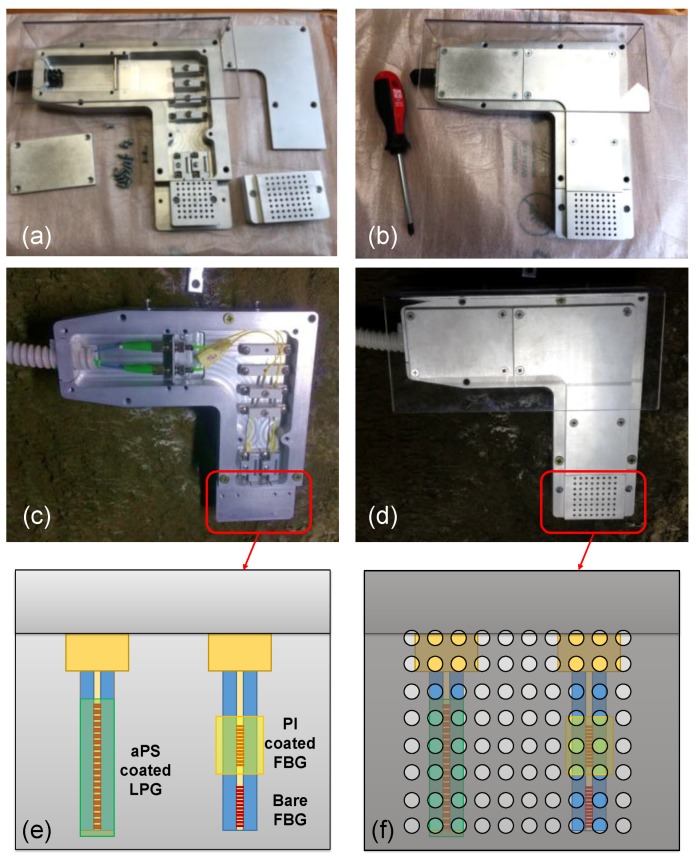
Photos of the realized package before (**a**,**b**) and after (**c**,**d**) its installation on the inner wall of the railway tunnel; (**e**,**f**) schematic (not to scale) of sensing area.

Some photos of the realized package are reported in Figure 7a,b. The package was installed on the inner wall of the railway tunnel, at 30 cm from the floor in a maintenance cavity located at 50 m far from the entrance (Figure 7c,d). Such a position was chosen considering that, in case of a leakage, liquefied petroleum gas occupies creeks and deposits on the floor, due to its higher density compared to air. Figure 7e,f show a schematic representation of the “sensing area” (~4 cm^2^), with the two single-ended fibers hosting the LPG-based gas sensor and the 2-FBGs array for temperature and humidity, respectively. The sensor package is connected with two approx. 500 m long single mode fiber patch-cords reaching the technical room with the interrogation unit. Finally, to avoid water infiltration inside the package, a Plexiglas canopy was also realized and mounted on the aluminum package.

### 2.4. Measuring Hardware and Software

The CU is located in the technical room of Porta Rufina Station. It involves the interrogation system for the acquisition of sensor spectra, controlled through a PC with custom-developed software, and aimed at acquisition as well as real-time data analysis. 

As anticipated, for the acquisition system, we selected the commercial HBM FS22 8-channel optical interrogator operating in the wavelength range 1500–1600 nm, having a maximum resolution of 1 pm and maximum acquisition rate of 1 S/s. To meet the requirements of the OPTOFER project, a custom LabView software for the real-time control of the interrogator unit as well as data analysis was developed by Migma srl. The software permits the acquisition of sensor spectra connected to the different optical channels and is able to track the resonance wavelength of both FBGs and LPGs. In addition, a programming interface permits sensor data analysis to compensate the effects of humidity and temperature changes and thus to estimate the gas concentration. Moreover, the software can notify an alarm to a remote server if the concentration exceeds a user-defined threshold. At any rate, these aspects lie beyond the aim of this manuscript.

## 3. In-Field Testing Results

The system was validated/tested in an operative railway scenario in Benevento (Italy) through different testing sessions. Each session was slightly longer than 24 h, across two different days. The sessions typically started in the mornings from 10:00 to 13:00, when the timetable of trains does not report any transits and was carried out until 13:00 of the following day. In particular, during the first day’s morning (Day 1), when it was possible to access the tunnel, the sensors were placed in the package (previously installed into the tunnel) and, after acclimatization time, we could simulate the presence of butane by injecting the gas in the proximity of the package. At about 13:00 of Day 1, the line was again operative, and thus we could monitor the sensor response during normal operations. During the second day’s morning (Day 2), additional gas tests were performed up to 13:00. During the night, the system was shut down for few hours. In the following, we report the main results of the performed in-field tests.

### 3.1. Testing Sessions

**Figure 8:**
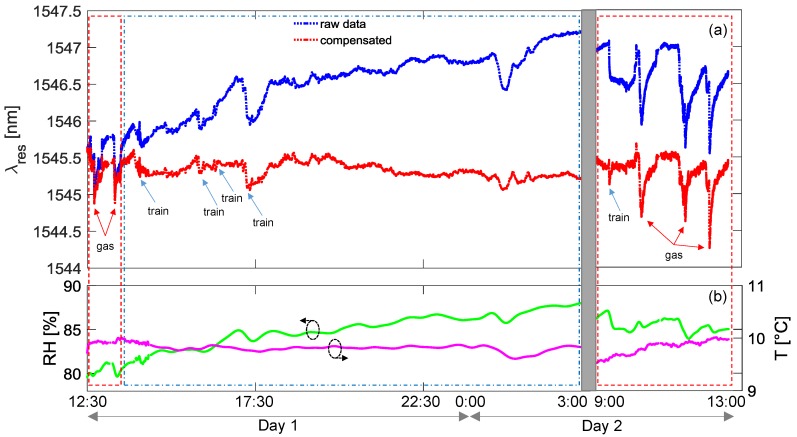
Results of complete in-field testing session in January 2017: (**a**) LPG sensor response; (**b**) temperature and humidity.

In Figure 8a,b, the data acquired during a testing session conducted in January 2017 are reported. Figure 8a shows the resonance wavelength of the gas sensor considering both raw data (blue line) and temperature and humidity compensated curve (red line). The environmental parameters are also reported in Figure 8b, as acquired from the array of FBGs. It can be clearly observed that average humidity was around 85% during this session, also probably due to rainy days. For convenience, during the tests with butane, the data were acquired every 3 s, whereas this interval was increased during long-term monitoring period to 30 s maximum. The overall figure can be divided into three time-windows: the red marked boxes identify the time intervals when the operator could access the tunnel to simulate the gas leakage (Day 1 and Day 2 mornings, respectively); the second interval (in the blue box) was restricted for any operation due to the train transits and was considered for long-term monitoring. Commercial butane gas was employed for sensor testing through injections of an air–butane mixture at a few centimeters from the sensors package. To this end, butane was first poured into a syringe where it was mixed with air in order to strongly decrease the concentration. Note that this procedure induces a very small gas leak and therefore does not represent a risk for safety. As one can observe, the exposition to butane produces negative spikes in resonance wavelength, i.e., a response similar to what was observed during the chamber characterization. A good reversibility of the response was measured as well when the gas concentration is dispersed after injection, as the aPS film permits the release of the gas molecules, with recovery times in 15–20 min.

We focus now on the time interval from 13:00 h of Day 1 to the following morning of Day 2 in Figure 8, when no leakage test could be performed due to train transits. In this period, the behavior of the (raw) resonance wavelength exhibits a slow monotonic increase of about 2.0 nm, with the presence of faster changes, principally due to the transit of trains (indicated by blue arrows in figure). The former effect is directly related to environmental changes and in particular to the relative humidity increasing from 81% to 88%, while the temperature variations were lower than 2 °C. This effect is quasi-completely compensated in the red curve reported in the same figure. Concerning the second effect, the train transits induce faster changes in the humidity sensor response and faster changes in temperature curve. However, from compensated data, it can be noted that in some cases the response of the sensor to trains is almost trivial, whereas in other cases it is more pronounced and not always completely compensated. We believe that this could be attributed to the exhaust gas from diesel-powered trains and thus could disappear in the case of electric trains. Additionally, another event resulting in a fast change of the raw resonant wavelength as well as the humidity curve is measured after midnight, which is not related to a train transit and is well compensated in the red curve.

**Figure 9:**
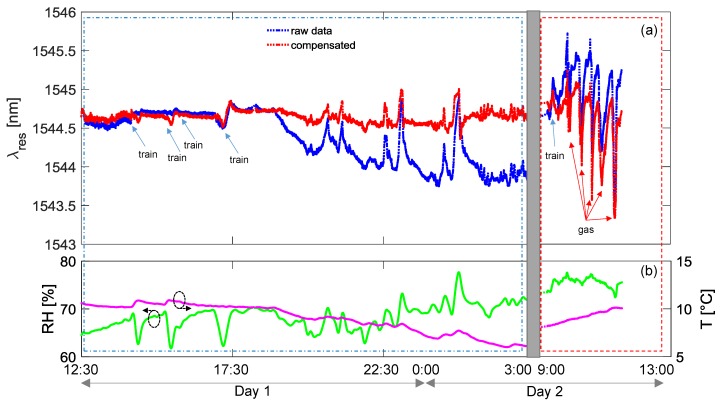
Results of complete in-field testing session in March 2017: (**a**) LPG sensor response; (**b**) temperature and humidity.

In Figure 9a,b, we report the results of a testing session conducted in March 2017, following the same above reported procedure. During this second test session, the recorded relative humidity was lower, at around 70%, due to the milder climate of the approaching spring season. The overall figure can be divided in two time-windows, where the resonance wavelength moved in a range 1.7 nm wide. The one marked by the red box identifies the morning of Day 2, when gas leakage was simulated and usual behavior was observed. Note that gas tests were not performed in the first day because the line was under maintenance and the access to tunnel was forbidden. Also, in this case, the blue box window refers to long-term monitoring and presents train transits. It is important noting that, during the evening/night, several quick changes of humidity level were recorded, as from Figure 9b green line, probably associated to climate events (e.g., strong gusts of wind in the tunnel). Consequently, quick changes in sensor response (blue line in Figure 9a) are measured. It is worth highlighting that even if such peaks were not completely removed after compensation, probably due to the different response time to humidity of the gas sensor and the reference sensors, their amplitudes (red line in Figure 9a) were significantly attenuated.

As an overall assessment, we can state that, during the different sessions, the resonance wavelength of LPG-based gas sensor moved in a range lower than 2 nm as a consequence of the changes occurring in all the mentioned perturbations. Concerning the red curves, where the environmental condition changes have been compensated, we found that the resonance wavelength moves in a range of ±0.3 nm (without considering gas leakages). As a result, we can assess that the uncertainty in the resonant wavelength detection induces an error in concentration of ±0.14 vol %, i.e., ±8% of the butane LEL. Moreover, it should be remarked that testing was conducted in the worst case, i.e., the trains are powered by diesel motors and thus their emissions can actually be a source of disturbance.

### 3.2. Gas Leakages Detection

**Figure 10:**
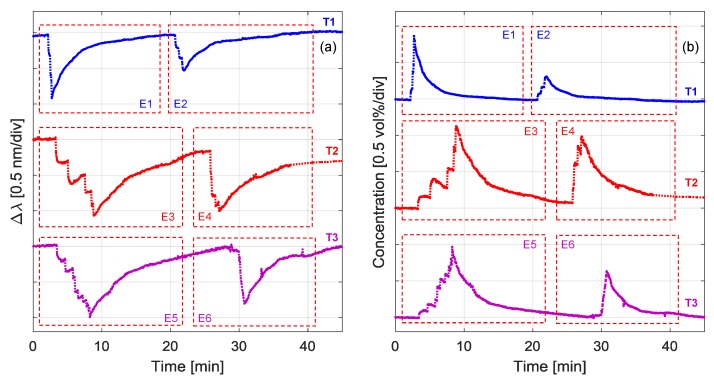
Results of in-field tests: (**a**) sensor response (after compensation); (**b**) corresponding butane concentration.

To better highlight the capability to detect gas leakages, Figure 10 compares the compensated curves of three representative tests (identified as T1, T2, and T3), conducted across time intervals of 45 min. These tests correspond to the acquired signal during the morning of three different sessions at the railway location. Compensation was well performed during the three periods, as the environmental conditions for the shorter time interval were obviously more stable than during the whole monitoring session. In particular, the temperature and humidity levels were around 10 °C and 80–85%, respectively, during these tests.

For simplicity, to highlight the expositions to butane, red boxes have been used in Figure 10a,b and labelled from E1 to E6. The gas expositions were performed by successive injections of air–butane mixtures. Consequently, the resonant wavelength moved from the baseline value by a step-wise increasing blue shift. It is worth noting that here the recoveries were in free evolution, in contrast to the chamber characterization setup used in laboratory. Nevertheless, a similar rising time of 2–5 min and recovery time of 15–20 min were measured, as reported in Section 2.1.

In Figure 10b, we have reported the corresponding butane concentrations, as calculated from the responses of Figure 10a and the characterization presented in Figure 3b. It appears clear that within the range 0–10 min (i.e., considering E1, E3 and E5), the concentration associated to the single exposition step is always reduced from T1 to T3, whereas the total concentration of the sequence is kept constant at ~1 vol %. Above this range (>10 min), the sensor signal returns to zero, allowing a second sequence of testing (i.e., E2, E4, and E6) characterized by a slightly lower accumulated concentration. As an overall consideration, it can be reported that during the different performed tests, a negative wavelength shift from 0.1 to 1.1 nm was revealed by the developed sensor, validating its reliability in detecting gas up to the maximum used concentration of 1 vol %.

## 4. Conclusions

In this work, the in-field demonstration of a fiber optic gas monitoring system, installed in an operative railway tunnel located in the south of Italy, was performed. As a target, the attention was focused on the butane, which is the main component of liquefied petroleum gas, since it is one of the dangerous species being transported with higher frequency (first in Italy) by railways lines.

The developed system comprises a sensing unit, installed directly in the tunnel, and a control unit, located in the station technical room. Customized software was developed in order to obtain the real-time measurements of gas concentrations, temperature and humidity levels in the tunnel in normal operative conditions of train transits. Specifically, during the validation tests, we have considered two main conditions: with the railway line under normal operation, and in the presence of controlled gas expositions. The results demonstrated that the system is always capable of detecting the traces of the gas during controlled expositions, whereas, during normal operation (i.e., in presence of trains transits), we found some false positives that can be quantified in ±8% of the LEL (slightly lower than the same value in commercial sensors).

It was demonstrated that, thanks to the advantages of all in-fiber technology, the system has the following important features: immunity to electromagnetic interferences, which are indeed a real problem in railways; operating in harsh conditions (even an explosive atmosphere); and operating at low temperature. The developed system could be easily scaled by introducing several sensing units due to easy multiplexing (in our case, up to eight measuring points are possible with the same setup) with the potential advantage of covering longer distances for monitoring purposes.

## References

[1] Kohl D. (2001). Function and applications of gas sensors. J. Phys. D Appl. Phys..

[2] Liu X., Cheng S., Liu H., Hu S., Zhang D., Ning H. (2012). A Survey on Gas Sensing Technology. Sensors.

[3] Hill K.O., Meltz G. (1997). Fiber Bragg grating technology fundamentals and overview. J. Lightwave Technol..

[4] Kersey A.D., Davis M.A., Patrick H.J., LeBlanc M., Koo K.P., Askins C.G., Putnam M.A., Friebele E.J. (1997). Fiber grating sensors. J. Lightwave Technol..

[5] Some S., Xu Y., Kim Y., Yoon Y., Qin H., Kulkarni A., Kim T., Lee H. (2013). Highly Sensitive and Selective Gas Sensor Using Hydrophilic and Hydrophobic Graphenes. Sci. Rep..

[6] Liu J., Sun Y., Fan X. (2009). Highly versatile fiber-based optical Fabry-Pérot gas sensor. Opt. Express.

[7] Zotti A., Zuppolini S., Giordano M., Zarrelli M., Borriello A., De Luca G. Optical aliphatic hydrocarbon gas sensor based on Titanium Dioxide thin film. Proceedings of the 2015 18th AISEM Annual Conference.

[8] Sun Y., Fan X. (2011). Optical ring resonators for biochemical and chemical sensing. Anal. Bioanal. Chem..

[9] Wu Y., Yao B., Zhang A., Rao Y., Wang Z., Cheng Y., Gong Y., Zhang W., Chen Y., Chiang K.S. (2014). Graphene-coated microfiber Bragg grating for high-sensitivity gas sensing. Opt. Lett..

[10] Yang M., Yang Z., Dai J., Zhang D. (2012). Fiber optic hydrogen sensors with sol–gel WO3 coatings. Sens. Actuators B Chem..

[11] Pilla P., Iadicicco A., Contessa L., Campopiano S., Cutolo A., Giordano M., Guerra G., Cusano A. (2005). Optical Chemo-Sensor Based on Long Period Gratings Coated With delta Form Syndiotactic Polystyrene. IEEE Photonics Technol. Lett..

[12] Pinto A.M.R., Lopez-Amo M. (2012). Photonic Crystal Fibers for Sensing Applications. J. Sens..

[13] Mishra S.K., Kumari D., Gupta B.D. (2012). Surface plasmon resonance based fiber optic ammonia gas sensor using ITO and polyaniline. Sens. Actuators B Chem..

[14] Usha S.P., Mishra S.K., Gupta B.D. (2015). Fiber optic hydrogen sulfide gas sensors utilizing ZnO thin film/ZnO nanoparticles: A comparison of surface plasmon resonance and lossy mode resonance. Sens. Actuators B Chem..

[15] Iadicicco A., Campopiano S., Giordano M., Cusano A. (2007). Spectral behavior in thinned long period gratings: Effects of fiber diameter on refractive index sensitivity. Appl. Opt..

[16] Cusano A., Iadicicco A., Pilla P., Contessa L., Campopiano S., Cutolo A., Giordano M. (2006). Mode transition in high refractive index coated long period gratings. Opt. Express.

[17] Cusano A., Iadicicco A., Pilla P., Cutolo A., Giordano M., Campopiano S. (2006). Sensitivity characteristics in nanosized coated long period gratings. Appl. Phys. Lett..

[18] Del Villar I. (2015). Ultrahigh-sensitivity sensors based on thin-film coated long period gratings with reduced diameter, in transition mode and near the dispersion turning point. Opt. Express.

[19] Pilla P., Trono C., Baldini F., Chiavaioli F., Giordano M., Cusano A. (2011). Giant sensitivity of long period gratings in transition mode near the dispersion turning point: An integrated design approach. Opt. Lett..

[20] Śmietana M., Koba M., Mikulic P., Bock W.J. (2016). Towards refractive index sensitivity of long-period gratings at level of tens of µm per refractive index unit: Fiber cladding etching and nano-coating deposition. Opt. Express.

[21] Wang T., Korposh S., James S., Tatam R., Lee S.-W. (2013). Optical fiber long period grating sensor with a polyelectrolyte alternate thin film for gas sensing of amine odors. Sens. Actuators B Chem..

[22] Cusano A., Iadicicco A., Pilla P., Contessa L., Campopiano S., Cutolo A., Giordano M., Guerra G. (2006). Coated long-period fiber gratings as high-sensitivity optochemical sensors. J. Lightwave Technol..

[23] Hromadka J., Tokay B., James S., Tatam R.P., Korposh S. (2015). Optical fibre long period grating gas sensor modified with metal organic framework thin films. Sens. Actuators B Chem..

[24] Hromadka J., Korposh S., Partridge M.C., James S.W., Davis F., Crump D., Tatam R.P. (2017). Multi-parameter measurements using optical fibre long period gratings for indoor air quality monitoring. Sens. Actuators B Chem..

[25] Topliss S.M., James S.W., Davis F., Higson S.P.J., Tatam R.P. (2010). Optical fibre long period grating based selective vapour sensing of volatile organic compounds. Sens. Actuators B Chem..

[26] Barnes J.A., Brown R.S., Cheung A.H., Dreher M.A., Mackey G., Loock H.-P. (2010). Chemical sensing using a polymer coated long-period fiber grating interrogated by ring-down spectroscopy. Sens. Actuators B Chem..

[27] Yang J., Zhou L., Huang J., Tao C., Li X., Chen W. (2015). Sensitivity enhancing of transition mode long-period fiber grating as methane sensor using high refractive index polycarbonate/cryptophane A overlay deposition. Sens. Actuators B Chem..

[28] Yang J., Che X., Shen R., Wang C., Li X., Chen W. (2017). High-sensitivity photonic crystal fiber long-period grating methane sensor with cryptophane-A-6Me absorbed on a PAA-CNTs/PAH nanofilm. Opt. Express.

[29] Falate R., Kamikawachi R.C., Müller M., Kalinowski H.J., Fabris J.L. (2005). Fiber optic sensors for hydrocarbon detection. Sens. Actuators B Chem..

[30] Monteiro-Silva F., Santos J.L., de Almeida J.M.M.M., Coelho L. (2018). Quantification of Ethanol Concentration in Gasoline Using Cuprous Oxide Coated Long Period Fiber Gratings. IEEE Sens. J..

[31] Esposito F., Zotti A., Ranjan R., Zuppolini S., Borriello A., Campopiano S., Zarrelli M., Iadicicco A. (2018). Single-Ended Long Period Fiber Grating Coated with Polystyrene Thin Film for Butane Gas Sensing. J. Lightwave Technol..

[32] Quero G., Consales M., Severino R., Vaiano P., Boniello A., Sandomenico A., Ruvo M., Borriello A., Diodato L., Zuppolini S. (2016). Long period fiber grating nano-optrode for cancer biomarker detection. Biosens. Bioelectron..

[33] Janczuk-Richter M., Dominik M., Roźniecka E., Koba M., Mikulic P., Bock W.J., Łoś M., Śmietana M., Niedziółka-Jönsson J. (2017). Long-period fiber grating sensor for detection of viruses. Sens. Actuators B Chem..

[34] Chiavaioli F., Baldini F., Tombelli S., Trono C., Giannetti A. (2017). Biosensing with optical fiber gratings. Nanophotonics.

[35] Esposito F., Ranjan R., Stăncălie A., Sporea D., Neguţ D., Becherescu N., Campopiano S., Iadicicco A. (2017). Real-time analysis of arc-induced Long Period Gratings under gamma irradiation. Sci. Rep..

[36] Stancălie A., Esposito F., Ranjan R., Bleotu P., Campopiano S., Iadicicco A., Sporea D. (2017). Arc-induced Long Period Gratings in standard and speciality optical fibers under mixed neutron-gamma irradiation. Sci. Rep..

[37] Stăncălie A., Sporea D., Neguţ D., Esposito F., Ranjan R., Campopiano S., Iadicicco A. (2018). Long Period Gratings in unconventional fibers for possible use as radiation dosimeter in high-dose applications. Sens. Actuators A Phys..

[38] Esposito F., Ranjan R., Campopiano S., Iadicicco A. (2018). Arc-Induced Long Period Gratings from Standard to Polarization-Maintaining and Photonic Crystal Fibers. Sensors.

[39] Esposito F., Ranjan R., Campopiano S., Iadicicco A. (2017). Experimental Study of the Refractive Index Sensitivity in Arc-induced Long Period Gratings. IEEE Photonics J..

[40] Iadicicco A., Ranjan R., Esposito F., Campopiano S. (2017). Arc-Induced Long Period Gratings in Polarization-Maintaining Panda Fiber. IEEE Photonics Technol. Lett..

[41] Ranjan R., Esposito F., Iadicicco A., Campopiano S. (2017). Arc-Induced Long Period Gratings in Phosphorus-Doped Fiber. IEEE Photonics Technol. Lett..

[42] Del Villar I., Matías I., Arregui F., Lalanne P. (2005). Optimization of sensitivity in Long Period Fiber Gratings with overlay deposition. Opt. Express.

[43] Ranjan R., Esposito F., Iadicicco A., Stancalie A., Sporea D., Campopiano S. (2016). Comparative Study of Long-Period Gratings Written in Standard and Fluorine-Doped Fibers by Electric Arc Discharge. IEEE Sens. J..

[44] Ranjan R., Esposito F., Campopiano S., Iadicicco A. (2017). Sensing Characteristics of Arc-Induced Long Period Gratings in Polarization-Maintaining Panda Fiber. IEEE Sens. J..

[45] Huang X.F., Sheng D.R., Cen K.F., Zhou H. (2007). Low-cost relative humidity sensor based on thermoplastic polyimide-coated fiber Bragg grating. Sens. Actuators B Chem..

